# Anti-Ro52 positivity is associated with progressive interstitial lung disease in systemic sclerosis—an exploratory study

**DOI:** 10.1186/s13075-023-03141-4

**Published:** 2023-09-04

**Authors:** Viggo Hamberg, Azita Sohrabian, Elizabeth R. Volkmann, Marie Wildt, Anna Löfdahl, Dirk M. Wuttge, Roger Hesselstrand, Göran Dellgren, Gunilla Westergren-Thorsson, Johan Rönnelid, Kristofer Andréasson

**Affiliations:** 1 Section of Rheumatology, Department of Clinical Sciences, Lund University, Lund, Sweden; 2 Department of Immunology, Genetics and Pathology, Uppsala University, Uppsala, Sweden; 3 Department of Medicine, Division of Rheumatology, University of California, Los Angeles, CA USA; 4 David Geffen School of Medicine, Los Angeles, CA USA; 5 Lung Biology, Department of Experimental Medical Science, Lund University, Lund, Sweden; 6 Department of Molecular and Clinical Medicine, Institute of Medicine, Sahlgrenska Academy, University of Gothenburg, Gothenburg, Sweden

**Keywords:** Systemic sclerosis, Interstitial lung disease, Ro52, Autoantibody, Biomarker

## Abstract

**Background:**

Interstitial lung disease (ILD) is the most common cause of death in patients with systemic sclerosis (SSc). Prognostic biomarkers are needed to identify SSc-ILD patients at risk for progressive pulmonary fibrosis. This study investigates autoantibodies measured in bronchoalveolar lavage (BAL) fluid and in serum in reference to the clinical disease course of SSc-ILD.

**Methods:**

Fifteen patients with new onset SSc-ILD underwent bronchoscopy. Autoantibody levels were analyzed using addressable laser bead immunoassay from BAL fluid and the serum. In a separate longitudinal cohort of 43 patients with early SSc-ILD, autoantibodies in serum were measured at baseline and pulmonary function tests were performed at least 2 times over the course of at least 2 or more years. Linear mixed effect models were created to investigate the relationship between specific autoantibodies and progression of SSc-ILD. Finally, lung tissue from healthy controls and from subjects with SSc was analyzed for the presence of the Ro52 antigen using immunohistochemistry.

**Results:**

Among SSc-ILD patients who were positive for anti-Ro52 (*N* = 5), 3 (60%) had enrichment of anti-Ro52 in BAL fluid at a ratio exceeding 50x. In the longitudinal cohort, 10/43 patients (23%) were anti-Ro52 positive and 16/43 (37%) were anti-scl-70 positive. Presence of anti-Scl-70 was associated with a lower vital capacity (VC) at baseline (-12.6% predicted VC [%pVC]; 95%CI: -25.0, -0.29; *p* = 0.045), but was not significantly associated with loss of lung function over time (-1.07%pVC/year; 95%CI: -2.86, 0.71; *p* = 0.230). The presence of anti-Ro52 was significantly associated with the loss of lung function over time (-2.41%pVC/year; 95% CI: -4.28, -0.54; *p* = 0.013). Rate of loss of lung function increased linearly with increasing anti-Ro52 antibody levels (-0.03%pVC per arbitrary units/mL and year; 95%CI: -0.05, -0.02; *p* < 0.001). Immunohistochemical staining localized the Ro52 antigen to alveolar M2 macrophages in peripheral lung tissue both in subjects with and without SSc.

**Conclusions:**

This study suggests that antibodies targeting Ro52 are enriched in the lungs of patients with new-onset SSc-ILD, linking Ro52 autoimmunity to the pulmonary pathology of SSc. Clinical and immunohistochemical data corroborates these findings and suggest that anti-Ro52 may serve as a potential biomarker of progressive SSc-ILD.

**Supplementary Information:**

The online version contains supplementary material available at 10.1186/s13075-023-03141-4.

## Introduction

Systemic sclerosis (SSc) is a systemic autoimmune disease characterized by progressive fibrosis of the skin and internal organs and has the highest cause-specific mortality among the rheumatic diseases [1]. SSc-ILD affects around 32–52% of patients with SSc, has a negative impact on health-related quality of life, and is the leading disease-related cause of death in patients with SSc [2–5]. SSc-ILD may progress at different rates, where some patients maintain stable lung function over time without any treatment, while other patients develop end-stage lung disease due to ILD, despite treatment with currently available therapy [6, 7]. Conventional immunomodulators such as cyclophosphamide and mycophenolic mofetil may attenuate disease progression and are commonly prescribed in SSc clinics [6, 8]. Other anti-rheumatic therapies, such as the biological agents, rituximab and tocilizumab, as well as the tyrosine kinase inhibitor nintedanib, have also been shown to modify disease progression [9–12]. All treatments come at the potential expense of adverse effects and treatments must be deliberately chosen and combined according to individual patient need [13]. Identifying patients at risk for progressive pulmonary fibrosis at the time of diagnosis may lead to earlier intervention with specific therapies aimed at averting irreversible lung damage [14, 15].

Autoantibodies are associated with distinct clinical phenotypes in SSc, including the presence of SSc-ILD [16]. For example, the autoantibody anti-topoisomerase 1, also known as anti-Scl-70, is associated with progressive SSc-ILD, and the autoantibody anti-Ro52 is associated with the presence of SSc-ILD and overall mortality in SSc [17–22]. The Ro52 antigen, also known as Tripartite motif-containing protein 21 (TRIM21), is an E3 ubiquitin ligase. Its purported function is to modulate immune reactions by ubiquitination of inflammatory mediators, and by extension, the development of autoimmune disease [23].

The majority of autoantibody studies in SSc measure autoantibodies in the sera. However, the measurement of autoantibodies in bronchoalveolar lavage (BAL) fluid may provide more direct insight into the pathobiology of ILD. In rheumatoid arthritis, relatively high concentrations of disease specific autoantibodies have been detected in BAL fluid in patients with signs of ILD [24]. To our knowledge, no prior studies have evaluated the presence of disease specific autoantibodies in BAL fluid from patients with SSc-ILD.

This study explores autoantibodies associated with SSc in reference to the pathogenesis and clinical disease course of SSc-ILD. The first objective was to compare the presence and relative enrichment of SSc-associated autoantibodies in BAL fluid with the serum in patients with relatively early SSc-ILD. The second objective was to determine whether the presence of anti-Ro52 in serum predicts progression of SSc-ILD in newly diagnosed patients. The overall hypothesis was that antibodies against Ro52 are associated with accelerated lung function decline in SSc-ILD. In an exploratory aim to further understand disease mechanism, we examined the local presence of the Ro52 antigen in lung samples from patients with and without SSc-ILD.

## Method

### Study participants

This study includes three separate cohorts. The *first cohort (“the BAL cohort”)* included patients with newly diagnosed SSc-ILD who were treatment naïve. These patients underwent bronchoscopy. The *second cohort (“the longitudinal cohort”)* included patients with SSc-ILD who underwent pulmonary function testing (PFT) twice or more during the first 5 years after disease onset. The *third cohort (“the IHC cohort”)* included patients with SSc-ILD, and healthy controls.

Patients with SSc-ILD fulfilled the following inclusion criteria: (1) 1980 American College of Rheumatology (ACR) preliminary classification criteria for SSc [25] and the 2013 ACR/European Alliance of Associations for Rheumatology (EULAR) classification criteria for SSc [26]; (2) ILD was based on interdisciplinary review of high resolution computed tomography (HRCT) and PFT results. Patients were excluded if they had pulmonary malignancy or were not able to understand written or spoken Swedish language.

Consecutive patients with SSc-ILD were invited to participate in the *BAL cohort* at time of SSc-ILD diagnosis, for a different study [27, 28]. Patients with suspected infection were excluded, as well as patients in whom bronchoscopy was associated with increased clinical risks. The subjects in this cohort have been discussed in detail elsewhere [27–29].

For the *longitudinal cohort,* patients with new onset SSc-ILD were selected from a prospective observational SSc cohort that included patients who had also been subject to extensive immunological characterization at the department of Immunology, Uppsala University Hospital. Subjects had to have performed their first PFT in 1999 or later and have a disease duration (defined as time from first non-Raynaud phenomenon [non-RP]) of 5 years or less at the time of cohort inclusion. Furthermore, these subjects had to have produced 2 PFTs or more at the same clinic during a follow-up time of at least 2 years. PFTs performed 5 years or more after disease onset were excluded.

For the *IHC cohort*, tissue from explanted lungs from SSc-ILD patients who underwent solid organ lung transplantation was analyzed using immunohistochemistry (IHC). Additionally, a group of subjects consisting of organ donors without pulmonary disease, whose lungs were donated for research purposes was included in this group.

### Study measurements

Age, sex, modified Rodnan Skin Score (mRSS) [30], % predicted VC, % predicted diffusing capacity for carbon monoxide (%pDLCO), and disease duration was recorded for all SSc-ILD patients. All SSc-ILD patients were classified as having diffuse cutaneous (dcSSc) or limited cutaneous (lcSSc) disease subtype [31]. For the BAL SSc-ILD cohort, these data were collected at the time of the bronchoscopy. For the longitudinal SSc-ILD cohort, these data were collected at the time of the first PFT. Immunomodulator use during the longitudinal follow-up period was noted (defined as ever use of mycophenolic acid, cyclophosphamide, rituximab, tocilizumab, nintedanib, azathioprine, methotrexate, and tumor necrosis factor inhibitors). Pulmonary arterial hypertension (PAH) diagnosed by right heart catheterization during the follow-up period was noted. Myositis, diagnosed by muscle biopsy during diagnosis was also noted.

### Collection of bronchoalveolar lavage fluid

Fifteen consecutive SSc-ILD patients naïve to immunomodulator treatment were included in the BAL cohort. BAL fluid was collected by a applying 100–150 ml of buffered saline divided into 3–4 installations, into a subsegmental bronchus of the middle lobe of the lung. The right middle lobe was chosen according to standard BAL methodology [32]. The volume of specimen recovered varied between 26–70 ml [29]. BAL fluid was stored frozen at -80 ℃ before laboratory analysis. BAL fluids were concentrated 20 × by usage of Amicon Ultra-4 Centrifugal Filters (Merck Millipore Ltd, Cork, Ireland) used according to the manufacturer’s instruction, and both non-concentrated and concentrated samples were analyzed.

### Pulmonary function test

PFTs were performed at the Skåne University Hospital in Lund using a Jaeger MasterScreen Body/Diff system (CareFusion, San Diego, CA, USA), and VC in liters was recorded. Percentage of predicted VC (%pVC) was calculated using patient age, body height and sex according to the reference equations for VC provided by the 2012 Global Lung Function Initiative [33].

PFTs were performed by trained hospital staff at the time of diagnosis, and then repeated yearly with some variation in frequency based on disease progression. All PFTs produced during the first 5 years after the first PFT were included. This time frame was chosen to limit observations to the pivotal years of lung function decline and to limit survival bias as previously described [14, 19].

### Immunological analyses

Quantification of anti-Scl-70, anti-centromere (ACA), anti-PmScl, and anti-U1RNP, anti-Ro52, anti-Ro60, anti-La/SSB autoantibody levels in BAL fluid specimen and patient sera was determined using addressable laser bead immunoassay (ALBIA; FIDIS connective tissue profile, Theradiag, Marne la Vallee, France) using the proprietary Solinium® software from Theradiag, with a modification to obtain more detailed quantitation in the low range for the BAL fluids. Patients were considered autoantibody positive for any antibody tested, if that specific autoantibody level was measured to be ≥ 40 arbitrary units/milliliter (AU/ml), according to the manufacturer’s instructions. Total IgG concentration (g/liter) in BAL fluid and serum were analyzed by ELISA as previously described [34].

In addition, anti-RNA polymerase III presence in serum was analyzed with a commercially available Fluorescense Enzyme ImmunoAssay (FEIA) in the 43 study participants that performed PFTs (Phadia EliA; MVZ Labor Prof. Seelig/Dr. Volkmann, Karlsruhe, Germany). Anti-nuclear antibody (ANA) patterns were determined according to current ICAP standards [35].

### Analyses of BAL fluid

*Relative autoantibody concentration* in both BAL fluid and serum was calculated as the quotient between a specific autoantibody and the total IgG antibodies, in BAL fluid and serum respectively.

*Autoantibody enrichment in the lungs* was calculated as a ratio between the relative autoantibody concentrations in BAL fluid and serum. This calculation was defined as:

$$Autoantibody\;enrichment\;in\;lungs=\frac{{}^{{BALF}_{autoantibody}}\!\left/ \!{}_{{BALF}_{Total IgG}}\right.}{{}^{{serum}_{autoantibody}}\!\left/ \!{}_{{serum}_{Total IgG}}\right.}$$

Differential cell counts from BAL fluid were performed on cytospin preparations and stained with the May–Grünewald–Giemsa. In total, 400 cells were counted by the same person.

### Immunohistochemistry of Ro52 in lung tissue

Formalin-fixed paraffin-embedded samples from peripheral lung tissue from the lower lung lobe were sectioned into 4 µm thick slices, deparaffinized and rehydrated according to standard IHC procedures. Heat-induced epitope retrieval at 6.0 pH (Dako Envision™Flex Target Retrieval solution) for 30 min was performed on a PT Tissue Link System (Histolab, Askim, Sweden). Monospecific and dual staining was performed with rabbit anti-Trim21 (anti-Ro52) monoclonal antibody (Cell signaling technology, Massachusetts, USA, cat no #92,043, dilution 1:500) and rabbit anti-CD206 polyclonal antibody (Abcam, Cambridge, UK, cat no. ab64693, dilution 1:4000). Tissue slides were treated with BLOXALL Endogenous blocking solution (Vector laboratories, Newark, CA, U.S, cat no. SP6000) for 10 min. After washing with Tris buffered saline (TBS), slides were incubated with normal horse serum 2.5% (Vector laboratories, cat no. 30022) for 20 min. Slides were then incubated overnight at + 4 °C with Trim21 antibody diluted in TBS and 2% bovine serum albumin (BSA). After washing with TBS-Tween-20 (0,05%) (TBS-T) slides were sequentially labeled with ImmPRESS® horse anti-rabbit IgG polymer kit, peroxidase (Vector laboratories, cat no. MP-7401) for 30 min, washed with TBS-T followed by ImmPACT® DAB EqV substrate kit, peroxidase (Vector laboratories, cat no. SK-4103) for 3 min. After washing, slides were incubated with normal horse serum 2.5% for 20 min and then incubated for 60 min with anti-CD206 antibody, the second primary antibody, diluted in TBS-BSA. Slides were washed and labeled with ImmPRESS®-AP horse anti-rabbit IgG Polymer kit alkaline phosphatase (Vector laboratories, cat no. MP-5401) for 30 min, washed with TBS-T, followed by treatment with Immpact®Vector® Red Substrate kit alkaline phosphatase (cat.no. SK-5105) for 10 min. Slides were counterstained with hematoxylin, dehydrated and mounted with Pertex mounting medium (Histolab, Askim, Sweden). The Images were obtained with a VS 120 virtual microscopy slide scanning system (Olympus, Tokyo, Japan) 20 × and analyzed with OlyVIA software 2.8 Olympus.

### Statistics

Baseline characteristics were compared using the χ^2^-test, Fisher’s exact test, or Mann–Whitney U test, as appropriate.

Longitudinal data was statistically analyzed using a linear mixed effects model (LMEM) with random by-patient intercepts and slopes for time, and %pVC as the outcome variable [19, 36]. Two models were specified, the first including anti-Ro52 presence or absence, as defined by concentration ≥ 40 AU/ml, and the second including the anti-Ro52 serum concentration as a continuous value. The covariates for the two models were otherwise identical and included other patient characteristics associated with the course of SSc-ILD (e.g., anti-Scl-70, disease type, and immunomodulator use, sex), as well as time-interaction terms [37]. In addition, predictor-follow-up time interaction effects were included as covariates in the model. In this study, we interpret this predictor-follow-up time interaction term as a predictor of decline of loss of lung function over time, a surrogate for SSc-ILD progression.

A *p*-value < 0.05 was considered statistically significant. All statistical analyses were performed using the statistical software R version 4.1.1 [38], using the lme4 package for LMEM model analysis [39].

### Ethics

This study uses data collected in a prospective, observational cohort from clinically indicated examinations that were performed independent of research purposes. Distal lung tissue derived from explanted lung tissue of healthy donors and lung transplant recipients were used in this study. Ethical approval was granted by the local ethics board (Lund University Ethics Committee 193/01, 2008/413, 2008/590, Swedish Ethical Review Authority: 2022–01221-02) and patients granted their written consent according to the Declaration of Helsinki.

## Results

### Enrichment of autoantibodies in BAL fluid

**Fig. 1:**
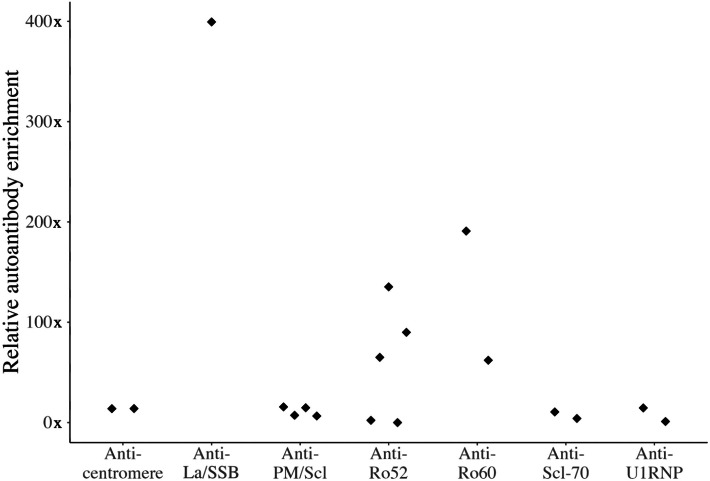
Enrichment of autoantibodies in BAL fluid in patients with new-onset SSc-ILD. Relative levels of the autoantibodies in BAL fluid compared to serum (y-axis). Only patients who tested positive for the specific antibody in serum are presented above

Among the 15 SSc-ILD patients who underwent bronchoscopy, 12 (80%) were seropositive for at least one of the autoantibodies studied (Table 1). The *autoantibody enrichment in lungs* was calculated for each antibody present in these patients. An enrichment of > 50 × in the BAL fluid as compared to serum was found for three autoantibody specificities. Anti-Ro52 was enriched in the lungs of 3/5 (60%) of anti-Ro52 seropositive patients, anti-Ro60 was enriched in 2/2 (100%) anti-Ro60 seropositive patients, and anti-La/SSB was enriched in the lungs of 1/1 (100%) anti-La/SSB seropositive patients (Fig. 1). No corresponding enrichment of anti-Scl-70, anti-U1RNP, anti-centromere, nor anti-PM/Scl was found. The aforementioned analysis was performed using unconcentrated BAL fluid. Corresponding analyses were performed with 20 × concentrated BAL fluid and yielded similar results (data not shown).

**Table 1:** Patient characteristics of patients subject to BAL

	Age	ANA^a^	SSc-associated antibodies^b^	SSc-subtype	mRSS	%pVC^c^	%pDLCO^d^	Disease duration (months)^e^	Sex	BAL cellularity, distribution (%)				
										Lymphocytes	Neutrophils	Eosinophils	Macrophages	Other
Pat 1	28	ANA positive. Nucleolar	Anti-PM/Scl	lcSSc	2	66	61	11	F	0	0	0	96	4
Pat 2	55	ANA positive. Centromere	Anti-Ro52 ACA	lcSSc	2	88	76	108	F	9	0	0	75	16
Pat 3	59	ANA positive. Speckled	Anti-Ro52 Anti-Ro60 Anti-La/SSB Anti-U1RNP	lcSSc	4	101	80	N/A	M	8	42	2	29	19
Pat 4	40	ANA positive. Nucleolar	Anti-PM/Scl Anti-U1RNP	dcSSc	6	80	42	24	M	13	10	5	57	15
Pat 5	55	ANA positive. Speckled	Anti-Scl-70	dcSSc	19	56	32	2	M	9	4	5	74	8
Pat 6	68	ANA positive. Speckled and homogenous	-	lcSSc	5	73	57	72	F	8	4	1	78	9
Pat 7	68	ANA positive. Speckled	Anti-Scl-70	lcSSc	9	57	45	50	F	12	20	0	54	14
Pat 8	68	ANA positive. Nucleolar	-	lcSSc	3	85*	88*	15	F	26	23	4	39	8
Pat 9	58	ANA positive. Nucleolar	Anti-Ro52 Anti-PM/Scl	dcSSc	34	62	28	11	M	7	6	7	57	23
Pat 10	56	ANA positive. Nucleolar	Anti-PM/Scl	dcSSc	20	70	81	40	F	9	28	2	35	26
Pat 11	67	ANA-negative	-	dcSSc	10	56	39	8	F	16	6	5	70	3
Pat 12	50	ANA positive. Homogenous	Anti-Ro52	dcSSc	15	93	62	12	F	5	1	1	93	0
Pat 13	70	ANA positive, pattern could not be defined	ACA	lcSSc	2	122	69	6	F	8	5	1	86	0
Pat 14	57	ANA negative	Anti-Ro60	dcSSc	19	95	62	36	M	6	5	4	84	1
Pat 15	46	ANA negative	Anti-Ro52	dcSSc	9	63	53	10	M	7	0	4	84	5

^a^Immunoflourescence pattern

^b^the following autoantibodies were tested *Anti-Ro52* Anti-Ro60 Anti-SSB/La, *Anti-U1RNP* Anti-PM/Scl, *ACA (anti-centromere)* Anti-Scl-70 (anti-topoisomerase)

^c^*%* predicted vital capacity

^d^% predicted diffusing capacity for carbon monoxide

^e^Since first first non-Raynaud’s phenomenon manifestation of SSc (months)

### Anti-Ro52 as a predictor of lung function decline in patients with SSc-ILD

Given the finding of anti-Ro52 antibody enrichment in the BAL fluid of SSc-ILD patients who were seropositive for anti-Ro52, we next examined whether anti-Ro52 measured in the sera was associated with progression of SSc-ILD.

Among the 43 patients who met our inclusion criteria for the longitudinal study (Table 2), the median number of PFTs per patient was 5 (interquartile range: 4–5), totalling 196 PFTs. The mean follow-up time was 3.7 years (SD 1.3). The first PFT was performed in 1999 and the last PFT was performed in 2020. PFT scores are presented longitudinally in Supplementary Fig. 1.

**Table 2:** Patient characteristics för SSc-ILD prospective group

Characteristic	Anti-Ro52 negative, *N* = 33 (77%)^a^	Anti-Ro52 positive, *N* = 10 (23%)^a^	Overall, *N* = 43^a^	***p*-value**^b^
Female	26 (79%)	9 (90%)	35 (81%)	0.656
Weight (kg)	70.8 (15.0)	61.0 (6.5)	68.5 (14.1)	**0.029**
Height (m)	1.68 (0.9)	1.63 (0.6)	1.67 (0.09)	0.185
Smoking^c^	12 (38%)	2 (20%)	14 (33%)	0.451
dcSSc	13 (39%)	3 (30%)	16 (37%)	0.719
Myositis	2 (6.1%)	2 (20%)	4 (9.3%)	0.226
PAH	3 (9.1%)	1 (10%)	4 (9.3%)	> 0.999
**Autoantibodies**				
Anti-Scl-70	13 (39%)	3 (30%)	16 (37%)	0.719
Anti-centromere	5 (15%)	1 (10%)	6 (14%)	> 0.999
Anti-RNA polymerase III	5 (15%)	2 (20%)	7 (16%)	0.656
**ANA pattern**^**d**^				
Homogeneous (AC-1)	12 (37%)	4 (40%)	16 (37%)	> 0.999
Centromere (AC-3)	4 (12%)	1 (10%)	5 (12%)	> 0.999
Large speckled (AC-5)	14 (42%)	8 (80%)	22 (51%)	0.069
Nucleolar (AC-8/9/10)	8 (24%)	2 (20%)	10 (23%)	> 0.999
Immunomodulator use^e^	30 (91%)	8 (80%)	38 (88%)	0.575
Baseline vital capacity (liters)	3.1 (0.8)	2.7 (0.6)	3.0 (0.8)	0.342
Baseline vital capacity (%predicted)	84 (16)	83 (17)	83.5 (16.0)	0.810
Disease duration at inclusion (years)^f^	1.8 (0.9)	1.8 (1.2)	1.8 (1.0)	0.499
Number of visits	5 (4, 5)	5 (4, 5)	5 (4, 5)	0.964
Follow-up time	3.7 (1.3)	3.6 (1.0)	3.7 (1.3)	0.518

^a^n (%), Mean (SD); Median (IQR)

^b^Pearson's Chi-squared test; Fisher's exact test; Mann–Whitney U test

^c^use ever

^d^ANA pattern as defined by ICAP

^e^use ever of mycophenolic acid, cyclophosphamide, rituximab, tocilizumab, nintedanib, azathioprine, methotrexate, tumor necrosis factor inhibitors; *dcSSc* diffuse cutaneous systemic sclerosis, *PAH* Pulmonary arterial hypertension

^f^disease onset defined as first non-Raynaud’s phenomenon symptom

In the longitudinal cohort 16/43 (37%) patients were anti-Scl-70 positive and 10/43 (23%) were anti-Ro52 positive. Of the anti-Ro52 positive patients, 3/10 (30%) were also anti-Scl-70 positive. Mean anti-Ro52 concentration was 110 AU/ml (SD ± 73). Patient characteristics did not significantly differ by anti-Ro52 status except by median body weight, which was higher in the anti-Ro52 positive group (71 kg vs 61 kg; *p* = 0.029; Table 2). In the anti-Ro52 positive group, 2/10 (20%) had concurrent myositis as compared to 2/33 (6.1%) in the anti-Ro52 negative group (*p* = 0.226).

### Anti-Ro52 positivity is associated with accelerated pulmonary function decline

Anti-Scl-70 seropositivity was associated with lower %pVC at time of diagnosis (at baseline, without time-interaction) (12.6%pVC; 95% CI: -25.0 – -0.29; *p* = 0.045; Table 3). Anti-Ro52 seropositivity was not significantly associated with lower %pVC at time of diagnosis (-1.25; 95% CI: -14.1 – 11.6; *p* = 0.844; Table 3). Neither disease cutaneous subtype, immunomodulator use, nor sex was associated with lower %pVC at time of diagnosis (Table 3).

**Table 3:** Predictive significance of patient characteristics on vital capacity

	**Antibody presence**			**Antibody concentration**		
*Predictors*	*Β*	*CI*	*p*	*Β*	*CI*	*p*
Intercept^a^	78.9	64.9 – 92.5	** < 0.001**	77.6	64.4 – 90.8	** < 0.001**
Follow-up time (years)^a^	1.23	0.03 – 2.42	**0.045**	1.10	0.12 – 2.09	**0.029**
**Change in baseline %predicted vital capacity at the time of SSc diagnosis**						
No immunomodulator use^bd^	11.3	-6.01 – 28.6	0.195	11.0	-5.95 – 28.0	0.197
Female sex^b^	12.7	-1.43 – 26.8	0.077	13.4	-0.21 – 27.0	0.053
dcSSc^b^	0.91	-11.4 – 13.2	0.882	3.2	-9.22 – 15.7	0.604
Anti-Scl-70^b^	-12.6	-25.0 – -0.29	**0.045**	-0.08	-0.15 – -0.01	**0.032**
Anti-Ro52^b^	-1.25	-14.1 – 11.6	0.844	0.01	-0.09 – 0.11	0.864
**Predicted change in %predicted vital capacity over time**						
No immunomodulator use * years^cd^	-2.33	-4.87 – 0.20	0.070	-1.68	-3.99 – 0.63	0.149
dcSSc * years^c^	-0.35	-2.14 – 1.43	0.691	-0.06	-1.73 – 1.61	0.944
Anti-Scl-70 * years^c^	-1.07	-2.86 – 0.71	0.230	-0.00	-0.01 – 0.01	0.483
Anti-Ro52 * years^c^	-2.41	-4.28 – -0.54	**0.013**	-0.03	-0.05 – -0.02	** < 0.001**

Predicted change in percentage of predicted vital capacity in patients with SSc-ILD, by patient characteristic, according to antibody presence and concentration. Presence of anti-Scl-70 predicted lower lung function at the time of diagnosis. Presence of anti-Ro52 autoantibody predicted significant decline of vital capacity over time in patients diagnosed with SSc-ILD. Higher anti-Ro52 concentration predicted significant decline of vital capacity over time in patients diagnosed with SSc-ILD

^a^Also included as random effects

^b^Time independent effect (%pVC at baseline)

^c^Time dependent effect (change in %pVC per year)

^d^use ever of mycophenolic acid, cyclophosphamide, rituximab, tocilizumab, nintedanib, azathioprine, methotrexate, tumor necrosis factor inhibitors; ILD, interstitial lung disease; *Β*, regression coefficient; CI, confidence interval; dcSSc, diffuse cutaneous systemic sclerosis; ICC, intraclass correlation coefficient

^*^indicates interaction term; *P*-values calculated using Kenward-Roger approximation of degrees of freedom

**Fig. 2:**
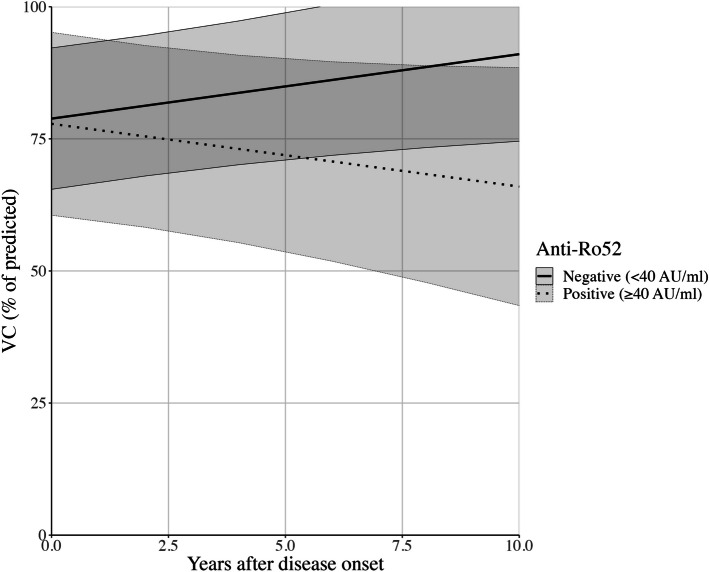
Anti-Ro52 positivity predicts decline in vital capacity. Lung function, expressed as percentage of predicted vital capacity (%pVC) over time, declines in anti-Ro52 seropositive systemic sclerosis patients with interstitial lung disease (-2.41%pVC/year; 95% CI: -4.28 – -0.54; *p* = 0.013). Prediction model using mixed model regression equation including anti-Ro52, anti-Scl-70, disease subtype, and immunomodulator use, with time-interaction terms, and sex; 95% confidence intervals indicated by shaded ribbons

Anti-Ro52 seropositivity was independently associated with loss of lung function over time in patients with SSc-ILD (-2.41%pVC/year; 95% CI: -4.28 – -0.54; *p* = 0.013), indicating that Ro52-positivity predicted accelerated loss of pulmonary function (Table 3; Fig. 2). No other predictors of SSc-ILD progression reached a level of statistical significance (Table 3).

### Higher anti-Ro52 level predicts accelerated pulmonary function decline

A second LMEM model was fitted to the patient data according to antibody levels. Higher anti-Scl-70 concentration was associated with lower %pVC at time of diagnosis (-0.08 per AU/ml; 95% CI: -0.15 – -0.01; *p* = 0.032**;** Table 3). Higher anti-Ro52 concentration was not significantly associated with lower %pVC at the time of diagnosis (0.01; 95% CI: -0.09 – 0.11; *p* = 0.864; Table 3).

**Fig. 3:**
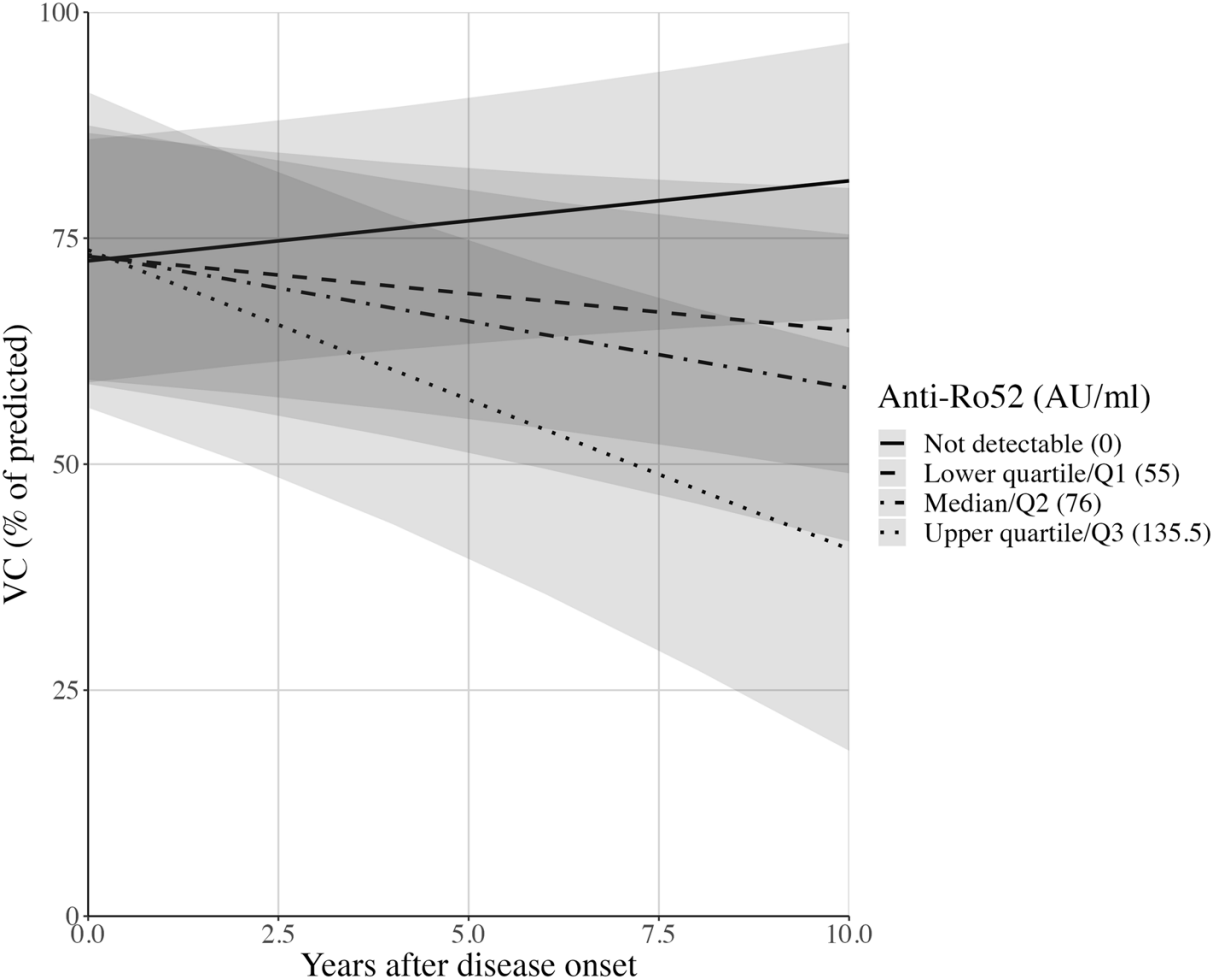
Anti-Ro52 concentration predicts decline in vital capacity. Lung function, expressed as percentage of predicted vital capacity (%pVC), decreases at a faster rate in patients with systemic sclerosis-associated interstitial lung disease and high anti-Ro52 levels (-0.03%pVC/year; 95% CI: -0.05 – -0.02; *p* =  < 0.001). Prediction model using mixed model regression equation including anti-Ro52 concentration, anti-Scl-70, disease subtype, and immunomodulator use, with time-interaction terms, and sex; 95% confidence intervals indicated by shaded ribbons

Increased anti-Ro52 concentration was associated with loss of lung function over time, indicating a progressive loss of pulmonary function in patients with the highest anti-Ro52 levels in serum (-0.03%pVC/year; 95% CI: -0.05 – -0.02; *p* =  < 0.001; Table 3, Fig. 3).

### Expression of Ro52 in peripheral lung tissue

**Fig. 4:**
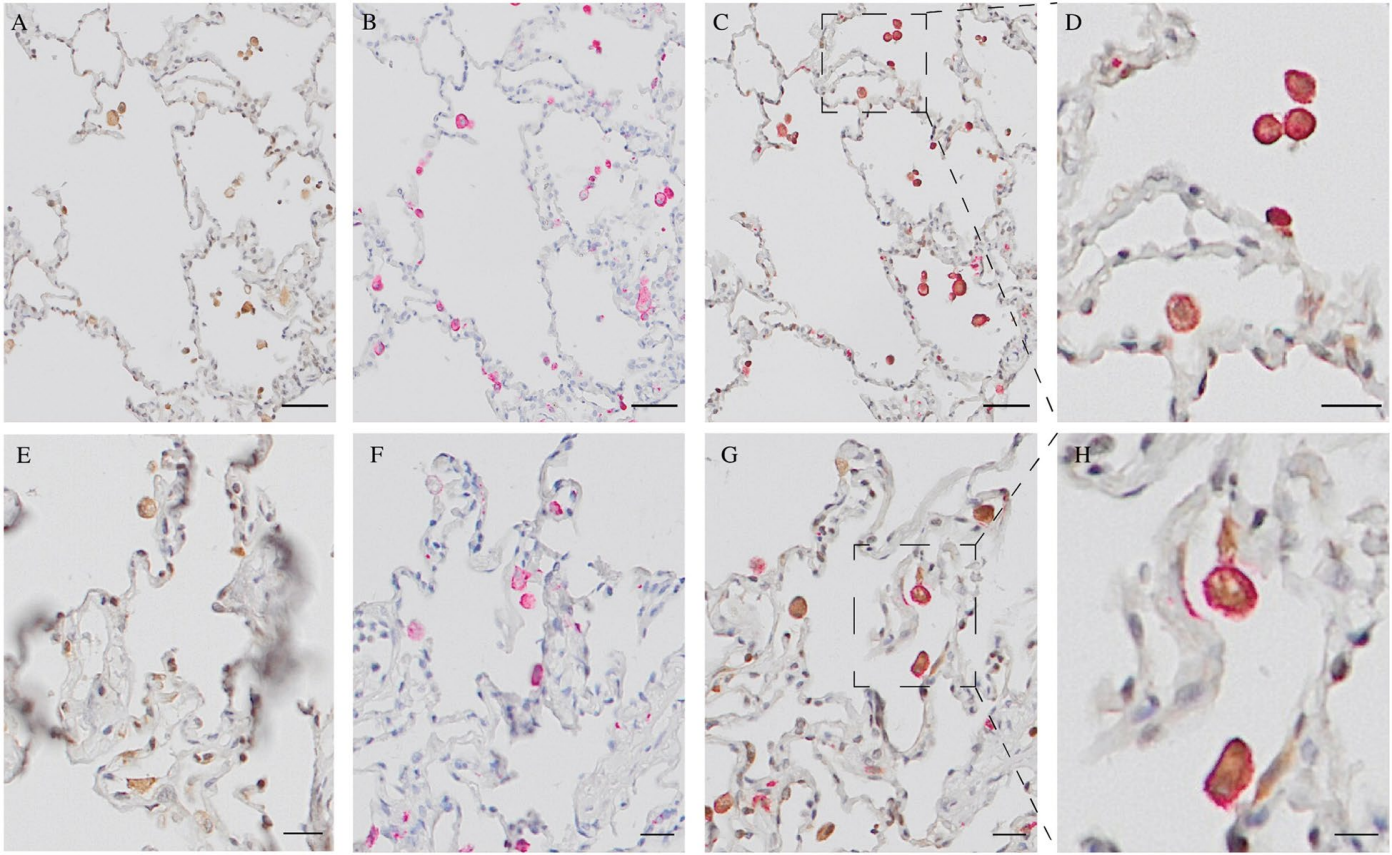
Ro52 present in peripheral lung tissue from healthy organ donors IHC from healthy peripheral lung tissue in donor 1 (**A**-**D**) and donor 2 (**E**–**H**) showing cellular localization of Ro52 (brown) in peripheral lung tissue (**A**, **E**) with distinct expression on alveolar macrophages (CD206; red; **B**, **F**). Co-staining with anti-CD206 and anti-Ro52 showed co-localization in alveolar M2 macrophages (**C**, **D**, **G**, **H**). Scale bar = 20 µm (A-C, E–G), 10 µm (**D**, **H**). Representative images from two healthy donors

Lung tissue samples from 6 male donors aged 25–68 with no known lung disease was analyzed with IHC. IHC staining located the Ro52 antigen to alveolar cells in healthy lung tissue (Fig. 4A, E). Co-staining with anti-CD206, a marker for M2 macrophages (Fig. 4B, F), indicated the expression of Ro52 in alveolar M2 macrophages (Fig. 4 C-D, G-H).

**Fig. 5:**
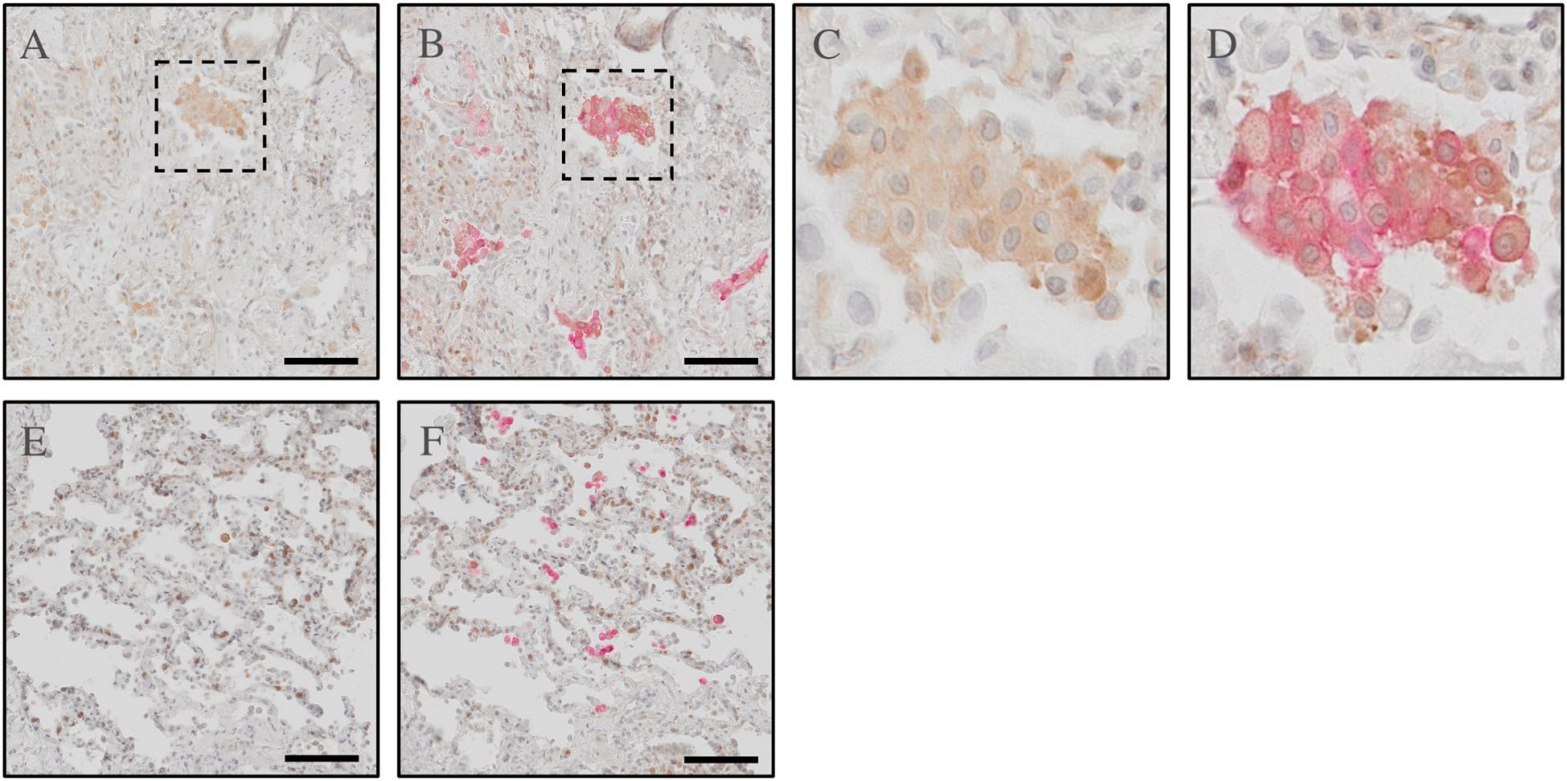
Ro52 present in peripheral lung tissue from SSc patients IHC from peripheral lung tissue from SSc patient 1 (**A**-**D** and SSc patient 2 (**E**–**F**) showing cellular localization of Ro52 (brown) in peripheral lung tissue (**A**, **C**, **E**). Co-staining with anti-CD206 and anti-Ro52 showed co-localization in alveolar M2 macrophages (**B**, **D**, **F**). Scale bar = 100 µm

In two subjects with a diagnosis of severe SSc-ILD, lungs were explanted following solid organ transplantation. In these lungs, the Ro52 antigen was expressed in a multitude of alveolar cells including M2 macrophages (Fig. 5).

## Discussion

In this study we have explored autoantibodies in relation to SSc-ILD and show that autoantibodies towards Ro52 are enriched in BAL fluid, and that the presence of these antibodies in serum is associated with progression of SSc-ILD.

Prior cross-sectional cohorts have evaluated the clinical significance of anti-Ro52 in patients with SSc. In these studies, the presence of anti-Ro52 was associated with the presence of ILD. To our knowledge, previous studies have not evaluated whether the presence of anti-Ro52 predicts progression of SSc-ILD, although one study demonstrated that anti-Ro52-positivity is associated with worse survival in patients with SSc [20]. In other disease states, including mixed connective tissue disease (MCTD) [40] and idiopathic inflammatory myositis, the presence of anti-Ro52 is associated with progression of ILD [41]. Anti-Ro52 has also been reported as a risk factor of developing ILD in primary Sjögren’s syndrome [42]. Moreover, in patients with antisynthetase syndrome, the presence of anti-Ro/SSA was more common in patients with severe ILD [43].

One longitudinal study evaluated whether the presence of anti-Ro (measured by immunodiffusion) was associated with lung function decline in patients with SSc, including both patients with and without ILD [19]. However, the immunodiffusion method employed by this study only precipitates anti-Ro60, and not anti-Ro52 [44, 45]. While Ro52 and Ro60 have historically been grouped together, they in fact have limited structural and functional homology with each other [46] and individually distinguish separate disease phenotypes [47]. Notably, the association between anti-Ro52 and Sjögren’s syndrome was not identified until 2018, in which anti-Ro52 and anti-Ro60 were analyzed separately in relation to ILD [42]. In this study we used ALBIA multiplexing technology, a method that accurately analyzes presence and semi-quantitative concentration of autoantibodies against Ro52 and Ro60 separately [47–49].

In addition to anti-Ro52 presence, we also explored the possible association between anti-Ro52 antibody levels and SSc-ILD progression (Fig. 4). Our results are in agreement with recent reports in antisynthetase syndrome were higher levels of anti-Ro52 are associated with more severe disease [50]. Similar findings have recently been presented in congenital heart block and anti-Ro52, further raising the questions if the concentration of Ro52-antibodies may be of also clinical importance [51]. Our results on anti-Scl70-levels are also in line with previous observations of an inverse relationship between anti-Scl-70 concentration and %VC [52].

Our study adds new perspective on the association between anti-Ro52 and lung fibrosis as we were able to demonstrate an accumulation of anti-Ro52 autoantibodies in the BAL fluid. This finding suggests a pathobiological link between the presence of anti-Ro52 antibody and the progression of SSc-ILD. Also, our BAL-results were carried out in patients with new-onset disease with relatively modest ILD. These findings are in line with previous reports suggesting that Ro-52 autoimmunity may precede development of ILD by several years [53]. Of interest, ILD without co-existing rheumatic disease has been linked to an increased prevalence of anti-Ro52 seropositivity [54].

Few studies have investigated enrichment of autoantibodies in BAL fluid of SSc patients. One study from 2014 demonstrated that anti-citrullinated protein antibodies (ACPAs) are enriched in BAL fluid of ACPA-positive patients with rheumatoid arthritis, and that these patients also exhibit lung abnormalities during early stages of disease [24]. In this study, it was hypothesized that early lung injury may initiate local molecular changes that generate immune responses in genetically susceptible individuals, subsequently causing systemic inflammatory disease. This hypothesis is of interest also to the SSc population considering our knowledge of inhaled environmental agents as a risk factor for SSc development [55, 56]. Taken together, the results of the present study raise the question if autoimmunity against Ro52 is initiated locally in the lungs of anti-Ro52 positive patients that develop SSc-ILD.

In our exploratory analysis, we demonstrated that Ro52 is naturally present in both healthy peripheral lung tissue and fibrotic peripheral lung tissue, and can be localized to M2 alveolar macrophages. M2 macrophages are involved in wound healing, and M2 macrophages are reported to be increased in both the skin and the peripheral blood of patients with SSc [57, 58]. Ro52 belongs to the tripartite motif protein family, which regulates the innate immune response including the antiviral immune response [23]. Ro52 functions as an E3 ubiquitin ligase, tagging proteins for proteasome degradation by binding to the Fc-region of immunoglobulins, that is in turn bound to the target protein on its antigen-target site [23, 49]. It is hypothesized that this property mediates an antiviral response that both degrades pathogens and regulates inflammatory mediators, such as the interferon regulatory factor (IRF) family [49, 59, 60]. Experimental studies have shown that Ro52 may regulate the production of autoantibodies and that inhibition of this antigen may result in progression of autoimmunity [61].

Limitations of this exploratory study include the number of patients included. While statistically significant, the results we present in this study need to be replicated and validated in a large and independent cohort. Another limitation of the study is that we limited the number of covariates in our statistical model to anti-Ro52, anti-Scl-70, disease type, sex, and immunomodulator use, and in doing so, may have failed to include other possible predictors of lung function decline, such as extent of pattern of lung involvement on HRCT, in the model. It is possible that the predictive power of anti-Ro52 may be mediated through some other predictors not included in our analysis. In addition, this study does not account for interaction effects between overlapping SSc-specific autoantibodies, or presence of anti-Ro60 autoantibodies. Our understanding of anti-Ro52 and SSc-ILD can be improved in future studies by including anti-Ro60 and anti-La, as the concentration of these were also increased in some BAL fluids. Another possible limitation is that the algorithm for determination of autoantibody levels using the proprietary FIDIS Theradiag software may underestimate very high antibody levels and thus overestimate the enrichment in BAL fluid. For that reason, and for follow-up studies, the Uppsala laboratory is currently developing new in-house algorithms for the comparison of autoantibody levels measured with ALBIA in different body compartments.

A strength of the study is its longitudinal design. Cross-sectional or survival analysis study designs are inadequate for drawing inferences about future ILD course [62, 63]. Another strength of the present study is that all PFTs were made at the same center.

If validated, our results may have clinical implications for the future assessment and management of SSc-ILD. In comparison to several previously presented biomarkers of progressive SSc-ILD, such as KL-6, CCL2, CCL18, and CXCL4, assessment of anti-Ro52 autoantibodies is a routine analysis at rheumatological centers [64]. Analysis of anti-Ro52 therefore has potential to be easily incorporated in the clinical risk stratification of SSc-ILD [65].

## Conclusions

In this study, we show that anti-Ro52 is enriched in BAL fluid in patients with new-onset SSc-ILD, linking anti-Ro52 autoimmunity to the pulmonary pathogenesis of SSc. In agreement with this initial finding, we also show that antibodies against Ro52 are associated with progressive SSc-ILD. If confirmed in larger validation studies, we suggest assessment of Ro52 to be incorporated in the risk stratification of new-onset SSc-ILD.

### Supplementary Information

**Additional file 1.**

## Data Availability

The datasets used and/or analysed during the current study are available from the corresponding author on reasonable request.

## Abbreviations

ACA: Anti-centromere autoantibodies
ACPA: Anti-citrullinated protein antibodies
ACR: American College of Rheumatology
ALBIA: Addressable laser bead immunoassay
ANA: Antinuclear antibody
AU/ml: Arbitrary units/ml
BAL: Bronchoalveolar lavage
BSA: Bovine serum albumin
dcSSc: Diffuse cutaneous systemic sclerosis
EULAR: European League Against Rheumatism
FEIA: Flourescense Enzyme ImmunoAssay
HRCT: High resolution computed tomography
IHC: Immunohistochemistry
ILD: Interstitial lung disease
IQR: Interquartile range
IRF: The interferon regulatory factor
lcSSc: Limited cutaneous systemic sclerosis
LMEM: Linear mixed effects model
MCTD: Mixed connective tissue disease
mRSS: Modified Rodnan Skin Score
PAH: Pulmonary arterial hypertension
PFT: Pulmonary function test
SD: Standard deviation
SSc: Systemic sclerosis
TBS: Tris Buffered Saline
TRIM21: Tripartite motif-containing protein 21(Anti-Ro52)
VC: Vital capacity
%pVC: Percentage of predicted vital capacity
%pDLCO: Percentage of predicted diffusing capacity for carbon monoxide
