# Supplementary figures and images for: Anti-Ro52 positivity is associated with progressive interstitial lung disease in systemic sclerosis—an exploratory study
Source: Arthritis Res Ther. 2023 Sep 4;25:162. doi: 10.1186/s13075-023-03141-4 (PMC10476305; doi:10.1186/s13075-023-03141-4)

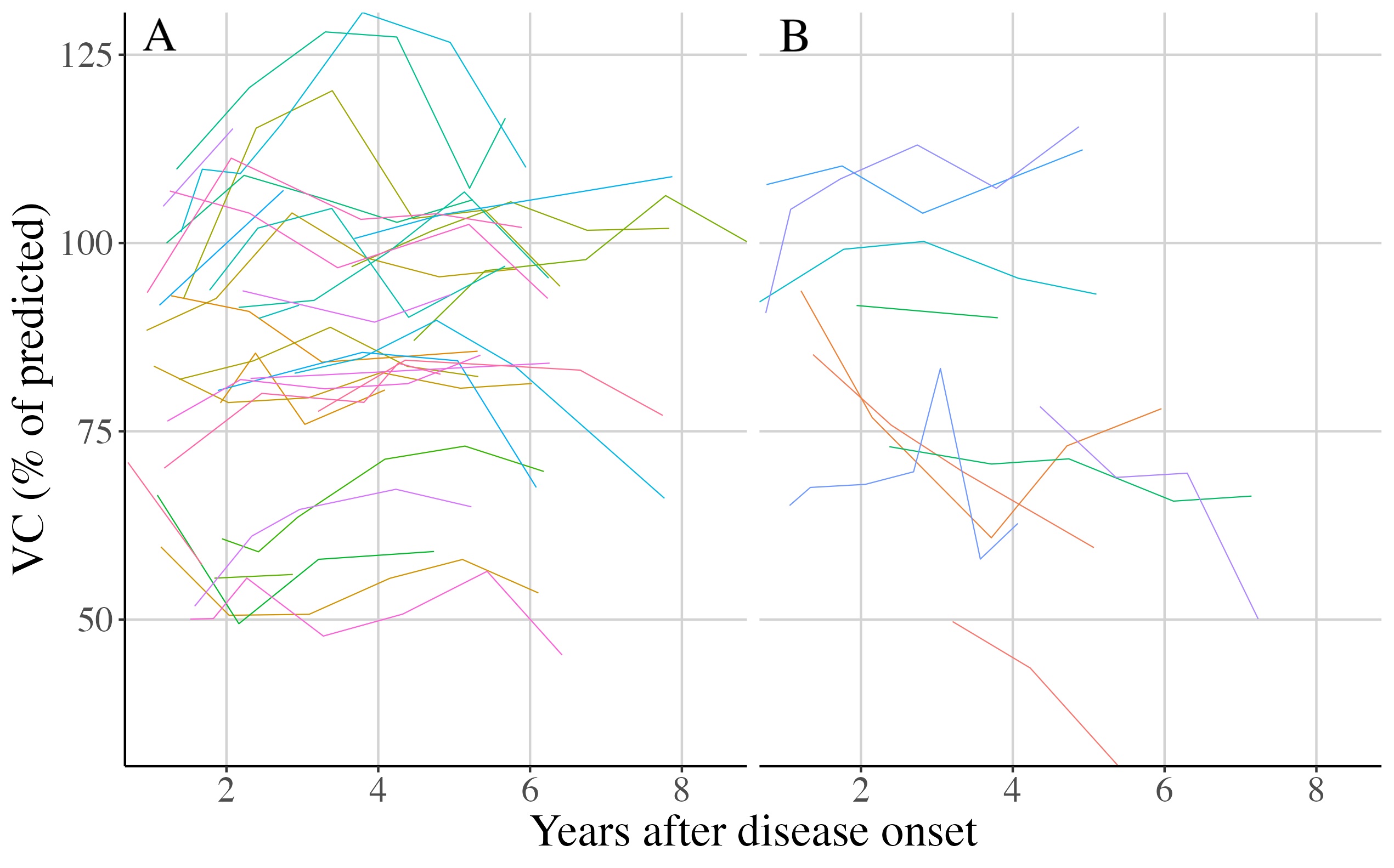

Supplement: Supplementary file 1 — Additional file 1. [file 13075_2023_3141_MOESM1_ESM.jpg]
